# Domestic violence’s impact on maternal–child relationship and child behavior: a nursing study from Egypt

**DOI:** 10.1186/s40359-025-03763-0

**Published:** 2026-01-20

**Authors:** Nadia Kasem Alaswad, Shymaa Mohammed Sayed Hassan, Hanan Azouz Abd Elhay, Mona R. Ahmed, Aml AbdElaal Mohamed Ali

**Affiliations:** 1 Pediatric Nursing Department, Faculty of Nursing, Cairo University, Cairo, Egypt; 2 Nursing Department, Faculty of Nursing, Irbid National University, Irbid, Jordan; 3 Psychiatric and Mental Health Nursing Department, Faculty of Nursing, Assiut University, Assiut, Egypt; 4 Pediatric Nursing Department, Faculty of nursing, Assiut University, Assiut, Egypt; 5 Maternal & Newborn Health Nursing, Faculty of Nursing, Assiut University, Assiut, Egypt; 6 Psychiatric Nursing and Mental Health Department, Faculty of nursing, South valley university, Qena, Egypt; 7 Primary care, Faculty of Nursing, Al-Ahliyya, Amman University, Amman, Jordan

**Keywords:** Domestic violence, Mother–child relationship, Child behavior, PTSD, Attachment theory, Egypt

## Abstract

**Background:**

Domestic violence is a global public health concern that negatively affects maternal mental health, parent–child relationships, and child behavioral outcomes. Attachment theory suggests that disruptions in maternal sensitivity and caregiving due to violence can compromise children’s emotional security and development.

**Aim:**

To evaluate the impact of domestic violence on maternal–child relationship quality and child behavior among pre-school and school-aged children in Egypt.

**Design:**

Descriptive correlational study.Setting: Maternal and Child Health Center, Assiut, Egypt.

**Participants:**

A convenience sample of 100 mothers with children aged 3–12 years.

**Methods:**

Data collection involved a Personal Data Questionnaire, the Severity of Violence Against Women Scale, the Child Behavior Checklist, and the Post-Traumatic Stress Scale for Family Violence. Statistical analyses included Pearson correlation, Chi-square, logistic regression, and ANOVA to examine associations and predictors.

**Results:**

Most mothers (58%) reported low levels of violence exposure. Severity of violence was strongly associated with maternal post-traumatic stress (*r* = .648, *p* < .001) and child behavioral problems (*r* = .650, *p* < .001) and negatively associated with mother–child relationship quality (*r* = –.185, *p* = .066). Regression analyses indicated that violence significantly predicted maternal PTSD and children’s clinical behavioral concerns, whereas demographic factors such as child age and maternal education moderated mother–child relationship outcomes.

**Conclusion:**

Exposure to domestic violence adversely affects maternal mental health and children’s behavioral outcomes, potentially compromising the mother–child relationship. These findings underscore the importance of interventions aimed at supporting maternal caregiving sensitivity, promoting secure parent–child attachment, and mitigating the effects of violence on family well-being in the Egyptian context.

**Supplementary Information:**

The online version contains supplementary material available at 10.1186/s40359-025-03763-0.

## Introduction

The family is the central unit of society, influencing the physical and mental well-being of its members and shaping children’s development [1, 2]. However, domestic violence (DV), particularly violence against women, remains a serious public health concern, including in Middle Eastern and North African countries (MENA) [3, 4]. The United Nations defines violence against women as “any act of gender-based violence that results in, or is likely to result in, physical, sexual, or mental harm or suffering to women, including threats of such acts, coercion or arbitrary deprivation of liberty, whether occurring in public or in private life” [5].

Domestic violence includes physical abuse, sexual assault, emotional abuse, financial control, and social isolation [6]. Despite laws in many countries aiming to protect women, enforcement is often inconsistent, and cultural norms may prevent women from reporting abuse or seeking support [7, 8]. In the Middle East and North Africa, approximately 35–40% of women report experiencing physical or sexual intimate partner violence during their lifetime [3, 4].

Exposure to DV has significant implications for children, including behavioral problems, emotional disturbances, and adverse developmental outcomes. Children who witness intimate partner violence are also at increased risk of morbidity and mortality, and adolescent exposure can contribute to long-term risky behaviors [6].

Recent evidence highlights the prevalence and multifaceted nature of domestic violence (DV) in Egypt. According to the Egypt Family Health Survey (EFHS) [7] conducted by the Ministry of Health and Population in collaboration with El-Zanaty and Associates and the United Nations Population Fund (UNFPA), approximately one-third of ever-married women aged 15–49 reported experiencing at least one form of spousal violence, including 25% who suffered physical abuse, 22% who endured psychological violence, and nearly 6% who experienced sexual assault [8]. Building on these findings, a 2024 study on DV identified three primary forms of DV; physical, psychological, and sexual and employed latent variable models to examine the influence of socioeconomic factors, such as education and employment status, on the likelihood of experiencing these abuses [9].

The current study is guided by Attachment Theory, which emphasizes the pivotal role of the caregiver(mother)–child relationship in shaping children’s emotional regulation, social competence, and behavioral adjustment. According to this perspective, consistent maternal responsiveness and sensitivity foster secure attachment, whereas disruptions in the caregiver’s availability or emotional stability may undermine the quality of the attachment bond and place children at greater risk for psychological and behavioral difficulties [10, 11].

Recent evidence supports the foundational role of the maternal–child attachment bond during early life stages. For example, La Rosa & Commodari [12] found that maternal psychological well-being, caregiving practices, and environmental stressors during pregnancy and postpartum significantly influence attachment quality, which then relates to emotional and behavioral outcomes in early childhood. This lends support to the present study’s focus on how DV and maternal post-traumatic stress might undermine the mother–child relationship and thereby impact child behavior.

A systematic review by Doroudchi et al. [13] reported that children exposed to domestic violence commonly experience a range of psychological complications, including internalizing problems such as depression, anxiety, and post-traumatic stress symptoms, as well as externalizing behaviors such as aggression and defiance. Similarly, a 2021 study [14] examining maternal exposure to domestic and community violence found that maternal victimization was directly associated with higher levels of depression and anxiety in children and indirectly linked to aggressive and withdrawn behaviors. Together, these findings highlight the significant emotional and behavioral impact of domestic violence on children’s psychological well-being.

In the context of DV, mothers are not only direct victims of abuse but may also experience compromised psychological well-being, including symptoms of post-traumatic stress. Such impairments can reduce their ability to provide sensitive caregiving, thereby weakening the maternal–child relationship. Consequently, children exposed to these dynamics are at higher risk of displaying maladaptive behaviors.

Evidence from Egypt underscores the impacts of DV not only on the victim but also on child behavior. For example, a recent survey by Mahrous et al. [14] demonstrated a strong association between maternal exposure to violence and child exposure and identified demographic factors that heighten this risk. These local findings align with global Attachment Theory based models [15], supporting our hypothesis that maternal psychological status and mother–child relationship quality mediate the effect of DV on children’s behavioral outcomes such as increased anxiety, fearfulness, irritability, sleep disturbances, social withdrawal, aggression, and difficulties with emotional regulation and peer relationships.

**Fig. 1:**
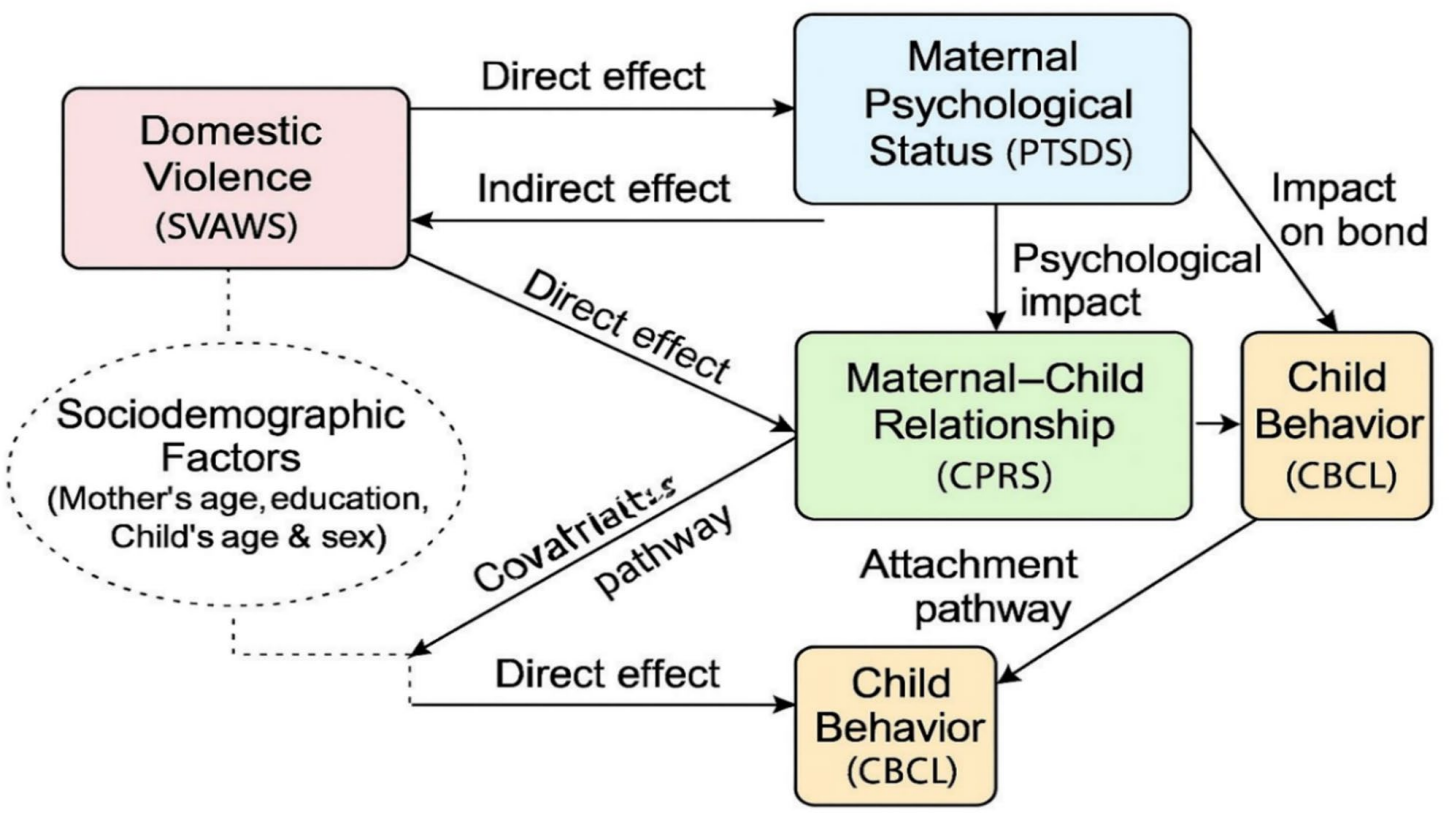
As shown in Figure 1, the study’s conceptual framework—guided by Attachment Theory—illustrates the hypothesized pathways through which DV may influence maternal psychological well-being, the maternal–child relationship, and ultimately child behavioral outcomes

The present conceptual framework (Fig. 1) illustrates these hypothesized pathways: DV affects maternal psychological status, which in turn influences the maternal–child bond and child behavior. Direct effects of DV on child behavior are also considered, alongside the potential influence of sociodemographic characteristics. This framework provides a theoretical rationale for examining the interrelated impacts of DV, maternal well-being, and maternal–child relationships on child behavioral outcomes within the Egyptian context.

Violence against women (VAW) encompasses all forms of gender-based violence that result in, or are likely to result in, physical, sexual, or psychological harm or suffering to women, whether occurring in public or private life. A major subset of VAW is intimate partner violence (IPV), which refers specifically to behaviors by a current or former intimate partner that cause physical, sexual, or psychological harm, including acts of physical aggression, sexual coercion, emotional abuse, and controlling behaviors (World Health Organization (WHO) [16]. IPV is the most common form of violence experienced by women globally and represents a key focus of domestic violence research due to its pervasive impact on maternal and child health.

As illustrated in Fig. 1, the prevalence of lifetime IPV remains high worldwide and is a critical issue in Egypt, where national surveys have reported that approximately one-third of ever-married women have experienced at least one form of spousal violence.

In Egypt, domestic violence (DV) remains a significant public health and social concern. According to the Central Agency for Public Mobilisation and Statistics (CAPMAS) 2021 data, 31% of currently or previously married women aged 15–49 reported having experienced one or more forms of intimate partner abuse (physical, sexual or psychological) by a spouse. Of these, 25.5% reported physical abuse and 22.2% reported psychological abuse [17].

Another large survey of ever-married women from the Egypt Demographic and Health Survey (EDHS) indicated 26.7% had experienced physical violence, 17.8% emotional violence and 4.6% sexual violence. These figures underscore the prevalence of DV in the Egyptian context and support the need for research and interventions tailored to maternal–child health services [18, 19].

Importantly, cultural and social norms in Egypt may affect how violence is defined, experienced and reported. For example, certain behaviors may not be labelled as “violence” within household or community narratives; reporting may be constrained by shame, family honor, economic dependency or limited recourse to formal support services [20]. Recognizing these factors is particularly relevant in nursing research and practice, as assessment, screening, disclosure and referral decisions are embedded within cultural and institutional contexts.

### Significance

Women’s physical and mental health is crucial, as it directly influences the well-being of their children and partners. Globally, the prevalence of lifetime intimate partner violence (IPV) varies by region, ranging from 20% in the Western Pacific, 22% in high-income countries and Europe, 25% in the Americas, to 33% in Africa [21].

Exposure to DV, particularly during pregnancy, can have significant adverse effects on maternal psychological well-being [22]. These effects may include psychosomatic disorders, substance use, difficulties in embracing the maternal role, and challenges in mother-infant attachment. Such consequences not only threaten maternal health but may also compromise newborn outcomes [23, 24].

Given these implications, the present study seeks to examine the impact of DV on maternal-child relationships, and children’s behavioral outcomes. Understanding this relationship is essential for informing interventions that promote maternal and child health in contexts affected by DV.

## Methods

### Aim of the study

This study aimed to evaluate the impact of domestic violence on maternal - child relationship and child behavior in pre and school-Age.

### Research questions

- Q1: What is the impact of domestic violence on the maternal–child relationship?
- Q2: How does domestic violence affect the psychological status of women?
- Q3: How does domestic violence influence child behavior in preschool and school-age children?
- Q4: Are sociodemographic characteristics associated with violence severity, maternal PTSD, or child outcomes?

### Research design

A descriptive correlational design was utilized.

### Theoretical framework Fig. 1

This study was guided by Attachment Theory, which posits that the quality of the emotional bond between a parent and child plays a pivotal role in the child’s social, emotional, and behavioral development [25, 26]. Exposure to DV may disrupt maternal emotional well-being and impair the mother’s capacity to maintain a secure and supportive relationship with her child. Such disruptions can contribute to maladaptive child behaviors and emotional difficulties. In line with this framework, the present study conceptualizes DV as an antecedent factor influencing maternal post-traumatic stress, which subsequently affects the maternal–child relationship. The quality of this relationship is, in turn, hypothesized to mediate the impact of violence on child behavioral outcomes.

### Setting

The research was done at the Maternal and Child Health Center in Assiut.

### Subjects

A convenience sampling technique was employed in this study. The research was conducted at the Maternal and Child Health Center in Assiut, Egypt, over a six-month period from March 2025 to August 2025. The target population consisted of women who had experienced domestic violence and their children.

A total of 100 mother–child pairs were recruited based on availability during the study period. Domestic violence exposure was determined using a self-report questionnaire method.

#### Inclusion criteria

- Mothers and their children who agreed to participate in the study (informed oral consent obtained).
- Children aged between 3 and 12 years.

#### Exclusion criteria

- Mothers with diagnosed severe mental illness as identified through self-report or medical record review.
- Children with known developmental disorders that is, conditions previously diagnosed by a physician or specialist and documented in the child’s health record (e.g., autism spectrum disorder, intellectual disability, or developmental delay).
- Mothers or children who refused to participate.

### Participant flow chart Fig. 2

During the study period (March–August 2025), a total of 118 mother–child pairs were screened for eligibility at the Maternal and Child Health Center in Assiut. 18 pairs were excluded:12 mothers declined participation, and 6 children had developmental disorders. The remaining 100 mother–child pairs met the inclusion criteria and were enrolled in the study. All participants provided oral informed consent, and no participants withdrew after enrollment. Thus, the final sample size was 100 pairs (mothers and their children).

**Fig. 2:**
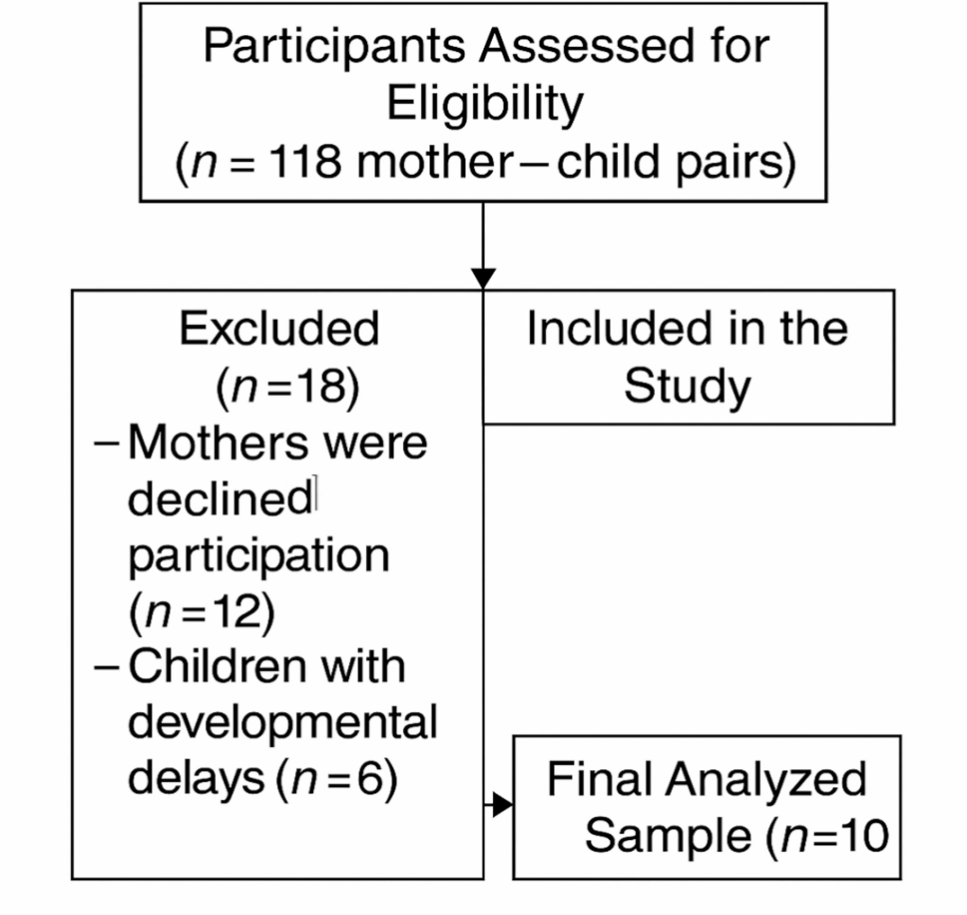
This figure illustrates the recruitment and participation process, including the number of mothers assessed for eligibility, those who declined participation, and the final sample included in the analysis *N* = 100

### Tools for data collection

Data were collected using the following standardized and researcher-developed instruments: the English version of the study’ used scales available in supplementary files.

#### Personal data questionnaire

This questionnaire was developed by researchers to collect demographic and background information about mothers and their children. It consisted of seven questions covering variables such as maternal and child age, child’s sex, parental educational level and occupation, as well as the number and frequency of violent incidents experienced by the mother.

#### Severity of Violence Against Women Scale (SVAWS)

The *Severity of Violence Against Women Scale* (SVAWS; Marshall [27]), is a standardized instrument designed to assess the severity, frequency, and intensity of physical, sexual, and psychological abuse in intimate partner relationships. The original scale consists of 46 items. For the purposes of this study, the instrument was culturally adapted to the Egyptian context after expert review by three specialists in maternal and community health nursing. Approximately half of the items were rephrased or removed because they were considered culturally sensitive or inappropriate. The final adapted version included 22 items rated on a 4-point Likert scale (1 = Never to 4 = Many times).

Abuse severity was categorized as low (22–50), moderate (51–79), and severe (80–88), based on the sample’s mean and standard deviation. This data-driven method aligns with previous cross-cultural adaptations of the SVAWS where full validation data were not available, thereby maintaining interpretive consistency with the original scale. Participants completed the questionnaire twice: once regarding experiences in the past year and once for their most recent previous partner. Scores were calculated by multiplying the frequency of each item by its severity weighting, with higher total scores indicating greater abuse severity. The original SVAWS demonstrated excellent internal consistency (Cronbach’s α = 0.98; [27]), supporting its reliability for both research and clinical applications.

Evidence from Middle Eastern populations highlights the relevance of assessing Intimate Partner Violence (IPV) severity in relation to maternal and child outcomes. Studies in Lebanon and Egypt have reported that higher IPV levels are associated with greater maternal stress, depression, and post-traumatic symptoms, which subsequently impair parenting practices and child emotional and behavioral adjustment [28, 29].

While the current study context is not classified as a conflict-affected region, certain socio-economic and cultural stressors such as poverty, unemployment, and gender-based power imbalances may produce comparable psychosocial dynamics within affected families. These contextual factors can intensify the psychological burden on mothers and disrupt parent–child relationships in ways similar to those reported in conflict-affected populations. In such contexts, IPV has been shown to disrupt parent–child relationships and elevate risks for child anxiety, aggression, and social withdrawal [30]. These findings reinforce the importance of employing culturally adapted and psychometrically sound tools such as the SVAWS to capture the nuanced experiences of IPV and their intergenerational impacts in the Egyptian and broader Middle Eastern context.

#### Post-Traumatic Stress Scale for Family Violence (PTSDS)

Maternal post-traumatic stress symptoms were assessed using the PTSDS, a 20-item self-report scale designed to evaluate PTSD symptoms related to family violence in accordance with DSM-5 criteria. Each item is rated on a 5-point Likert scale (0 = Not at all to 4 = Extremely), yielding total scores ranging from 0 to 80. Symptom severity is categorized as: no symptoms (0), mild (1–20), moderate (21–45), moderate-to-severe (46–72), and severe (73–80). The scale demonstrated excellent internal consistency Cronbach’s α = 0.91 [31].

The PTSDS has been widely used in research exploring the impact of maternal trauma on child outcomes in conflict-affected populations. For example, Peltonen et al. [32] found that maternal PTSD in Syrian refugee families predicted significant emotional and behavioral changes in children. Similarly, Nehring et al. [33] highlighted the utility of the Child Behavior Checklist (CBCL) in screening for behavioral problems linked to maternal trauma in Syrian-origin families. These findings support the use of the PTSDS as a reliable tool for investigating the pathway between maternal PTSD and child behavioral outcomes in regional, trauma-affected contexts.

#### Child Behavior Checklist (CBCL)

Children’s behavioral and emotional problems were assessed using the Arabic version of the Child Behavior Checklist (CBCL). For children aged 3–5 years, the CBCL/1½–5 form was administered, while for those aged 6–12 years, the CBCL/6–18 form was used [34, 35]. Both instruments are scored on a 3-point Likert scale (0 = “not true”, 1 = “somewhat or sometimes true”, 2 = “very true or often true”).

In this study, the Arabic version validated by Seleem et al. [36] for ages 6–18 and previously applied in Egyptian samples Abdelmonem et al., [37] was used. The instruments were culturally adapted and reduced to 60 items across the two age versions, while maintaining the core subscale. Scores were classified into normal, borderline, or clinical ranges, consistent with CBCL guidelines, with higher scores indicating greater behavioral and emotional difficulties.

Psychometric support: The Arabic CBCL/6–18 demonstrated good internal consistency and structural validity in Egyptian children. For the younger version, the Arabic-translated CBCL 2–3 has previously shown strong reliability in the UAE, with Cronbach’s α ≈ 0.93 for the total score and 0.70–0.88 across subscales [37]. In the current study, internal consistency was strong, with Cronbach’s α ≈ 0.94 for the total CBCL score.

#### Child–Parent Relationship Scale (CPRS)

The *Child–Parent Relationship Scale* (CPRS) is a 15-item self-report instrument designed to assess parents’ perceptions of their relationship with their children. Items are rated on a 5-point Likert scale ranging from 1 (Definitely does not apply) to 5 (Definitely applies). The scale comprises two subscales: Conflict (8 items), reflecting negative aspects of the parent–child relationship, and Closeness (7 items), capturing warmth, affection, and open communication. Higher scores indicate stronger relationship quality. CPRS has demonstrated acceptable reliability, with Cronbach’s α = 0.76 [38].

While Egypt is not considered a conflict-affected or refugee context, families experiencing domestic violence may be exposed to chronic psychosocial stressors that mirror some of the emotional and relational disruptions seen in trauma-affected populations. Previous studies in Middle Eastern refugee and conflict-affected families have shown that maternal trauma can significantly influence parent–child relationship quality. For example, Barnes [39] found that maternal trauma among Middle Eastern refugee families was associated with increased parenting stress and diminished emotional availability, resulting in lower child attachment security. Similarly, research by Maloney et al. [40] in Palestinian families indicated that maternal trauma exposure negatively impacted adolescent emotional adjustment, illustrating the intergenerational effects of stress and trauma. Recognizing these parallels underscores the relevance of the CPRS in capturing relationship dynamics among Egyptian families affected by domestic violence, where ongoing stress and exposure to violence may similarly impair emotional closeness and increase parent–child conflict.

### Ethics approval and consent to participate

Ethical approval was obtained from the Scientific Research Ethics Committee, Faculty of Nursing, Assiut University, Egypt (Approval Code: 1120251104). Permission to conduct the study was also granted by the Manager of the Maternal and Child Health Center in Assiut. Prior to data collection, all participating women received a clear explanation of the study’s purpose, procedures, potential benefits, and the voluntary nature of participation. Informed oral consent was obtained from each mother on behalf of herself and her child, in accordance with the approval of the ethics committee.

Participants were assured that no harm would result from their participation and that they had the right to withdraw from the study at any time without negative consequences. Confidentiality and anonymity were strictly maintained, and data were used solely for research purposes. This study adhered to the ethical principles outlined in the Declaration of Helsinki: (http://www.wma.net/en/30publications/10policies/b3/index.html) [41] regarding research conducted on humans and human data.

### Data security

All data were handled in strict confidence. Each participant was assigned a unique code, and no identifying information was included in the dataset. Electronic files were password-protected and stored on a secure computer accessible only to the research team. Hard copies of questionnaires were locked in secured cabinets at the Faculty of Nursing, Assiut University. Data will be retained for five years after study completion and then permanently destroyed. The dataset was used solely for research purposes and was not shared with unauthorized individuals.

### Data Availability

The datasets generated and/or analyzed during the current study are not publicly available due to privacy and ethical restrictions but are available from the corresponding author on reasonable request and with approval of the Faculty of Nursing, Assiut University Ethics Committee.

### Pilot study

A pilot pretest was conducted on 10% of the total sample to evaluate the clarity, feasibility, and cultural appropriateness of the data collection tools. This pretest focused on assessing the comprehensibility and relevance of questionnaire items rather than testing the full study procedures. It complemented the expert validity assessment carried out by five professionals in psychiatric and mental health nursing and pediatric nursing, ensuring both theoretical soundness and practical applicability of the instruments. The pilot confirmed that the questionnaires were understandable, acceptable, and did not impose unnecessary burden on participants. As no major modifications were required, these participants were included in the final study sample.

### Procedure

The fieldwork was conducted over a six-month period, from March 2025 to August 2025, and comprised two main phases:

#### Preparatory phase

The researchers conducted a comprehensive review of relevant literature to establish a strong theoretical foundation for the study. Based on this review, the study tools were developed, adapted, and assessed for content validity by a panel of expert reviewers in psychiatric and mental health nursing and pediatric nursing. The experts evaluated the instruments for clarity, relevance, representativeness, and cultural appropriateness to ensure they accurately reflected the intended constructs. Following this step, a pilot pretest was conducted to further confirm the clarity, feasibility, and cultural suitability of the tools before initiating the main data collection.

#### Implementation phase

Following ethical approval from the Faculty of Nursing, Assiut University, and receipt of an official letter of permission from the Dean of the Faculty to the Manager of the Maternal and Child Health Center in Assiut, the researchers commenced data collection. Eligible women exposed to DV and their children were approached individually at the Center. The researchers explained the study objectives, procedures, voluntary nature of participation, and confidentiality measures before obtaining oral informed consent. Data collection sessions were conducted three days per week, with each interview lasting approximately 30–60 min depending on the participant’s availability. Both mothers and their children were engaged in the process using the validated study instruments.

Data collection activities Table 1 were distributed across six months to ensure systematic progression and adequate participant recruitment. The preparatory phase, including literature review and tool development, was completed in March 2025, followed by expert validation and pilot testing in early April. Recruitment of eligible women and their children began in April and continued through May, overlapping with the start of data collection to optimize efficiency. Data collection was carried out consistently three days per week from April to August 2025, with each interview lasting 30–60 min. Final verification and completion of the dataset were performed in July and August to ensure accuracy and completeness before analysis.

**Table 1:** The data collection timeline

Study phase/Activity	March 2025	April 2025	May 2025	June 2025	July 2025	August 2025
Literature review and tool preparation	✔					
Expert validation of tools	✔					
Pilot study		✔				
Recruitment of participants		✔	✔			
Data collection (interviews with mothers/children)		✔	✔	✔	✔	✔
Data completion and verification					✔	✔

(Table 1) shows the sequential phases of tool preparation, pilot testing, recruitment, and data collection. This complements the participant flow diagram (Fig. 2), which illustrates the number of women and children screened for eligibility, excluded, and finally included in the study.

### Statistical analysis Table 2

Data were collected, coded, revised, and entered into the Statistical Package for the Social Sciences (IBM SPSS Statistics, version 22). Descriptive statistics were used to summarize the data: frequencies and percentages for categorical variables and means with standard deviations for continuous variables.

**Table 2:** Planned statistical analyses by research question: summary of the statistical tests selected to address each research question, showing the study variables involved, the type of analysis applied, and the purpose of each test

Research question	Variables involved	Statistical test(s) applied	Purpose
1. What is the impact of DV on the maternal–child relationship?	Independent: Severity of violence (SVAWS^1^ score) Dependent: Mother–child relationship (CPRS^2^ score)	Pearson correlation Multiple linear regression	To assess the relationship and identify predictors of relationship quality
2. How does DV affect the psychological status of women?	Independent: Severity of violence (SVAWS score) Dependent: PTSD^3^ symptoms (PTSDS score)	Pearson correlation Multiple linear regression	To determine the association and predictive effect of violence severity on maternal PTSD
3. How does DV influence child behavior in preschool and school-age children?	Independent: Severity of violence (SVAWS score), maternal PTSD Dependent: Child behavior (CBCL^4^ scores; normal/borderline/clinical)	Pearson correlation Chi-square Logistic regression	To explore associations and examine predictors of child behavior outcomes
4. Are sociodemographic characteristics associated with violence severity, maternal PTSD, or child outcomes?	Independent: Mother’s age, education, occupation, child’s age, sex Dependent: SVAWS, PTSDS, CBCL, CPRS scores	Chi-square ANOVA/t-test Multiple regression	To identify possible confounding or moderating effects of demographic factors

^1^ *Severity of Violence Against Women Scale*

^*2*^ *Child–Parent Relationship Scale*

^*3*^ *Post-Traumatic Stress Scale for Family Violence*

^*4*^ *Child Behavior Checklist*

For group comparisons, the Chi-square test (χ²) was used to examine associations between categorical variables. One-way analysis of variance (ANOVA) or independent-sample *t* tests were applied to compare mean differences across groups when appropriate. Pearson’s correlation coefficient (*r*) was employed to assess relationships between continuous study variables (e.g., severity of violence, PTSD symptoms, child behavior scores, and mother–child relationship scores).

To identify predictors of key outcomes, regression analyses were conducted. Multiple linear regression was used to assess the influence of DV severity and maternal PTSD symptoms on continuous outcomes such as child behavior scores and mother–child relationship scores. Logistic regression was applied when outcomes were categorical (e.g., child behavior classified as normal, borderline, or clinical). Potential confounding variables (e.g., mother’s age, educational level, and child’s age) were adjusted for in the regression models.

The level of statistical significance was set at *p* <.05, with *p* <.01 considered highly significant. All tests were two-tailed, and 95% confidence intervals were reported where applicable.

As shown in Table 3, the children’s mean age was 9.44 ± 3.48 years, with slightly more females (56%). Mothers’ mean age was 32.0 ± 4.40 years. Most parents held university degrees, all fathers were employed, and variation was mainly observed in maternal occupation (59% housewives, 41% employed).

**Table 3:** Sociodemographic data of the study sample (*n* = 100)

Variable	*N*	%
Child Age		
Mean ± Std	**9.44 ± 3.48 **	
Child gender		
Male	44	44
Female	56	56
Mother' s age		
Mean ± Std	**32.0** **± 4.40**	
Mother' s education		
preparatory	1	1
Secondary	22	22
University	77	77
Father' s education		
Secondary	22	22
University	78	78
Mother' s occupation		
Housewife	59	59
working	41	41
Father' s occupation		
working	100	100

**Fig. 3:**
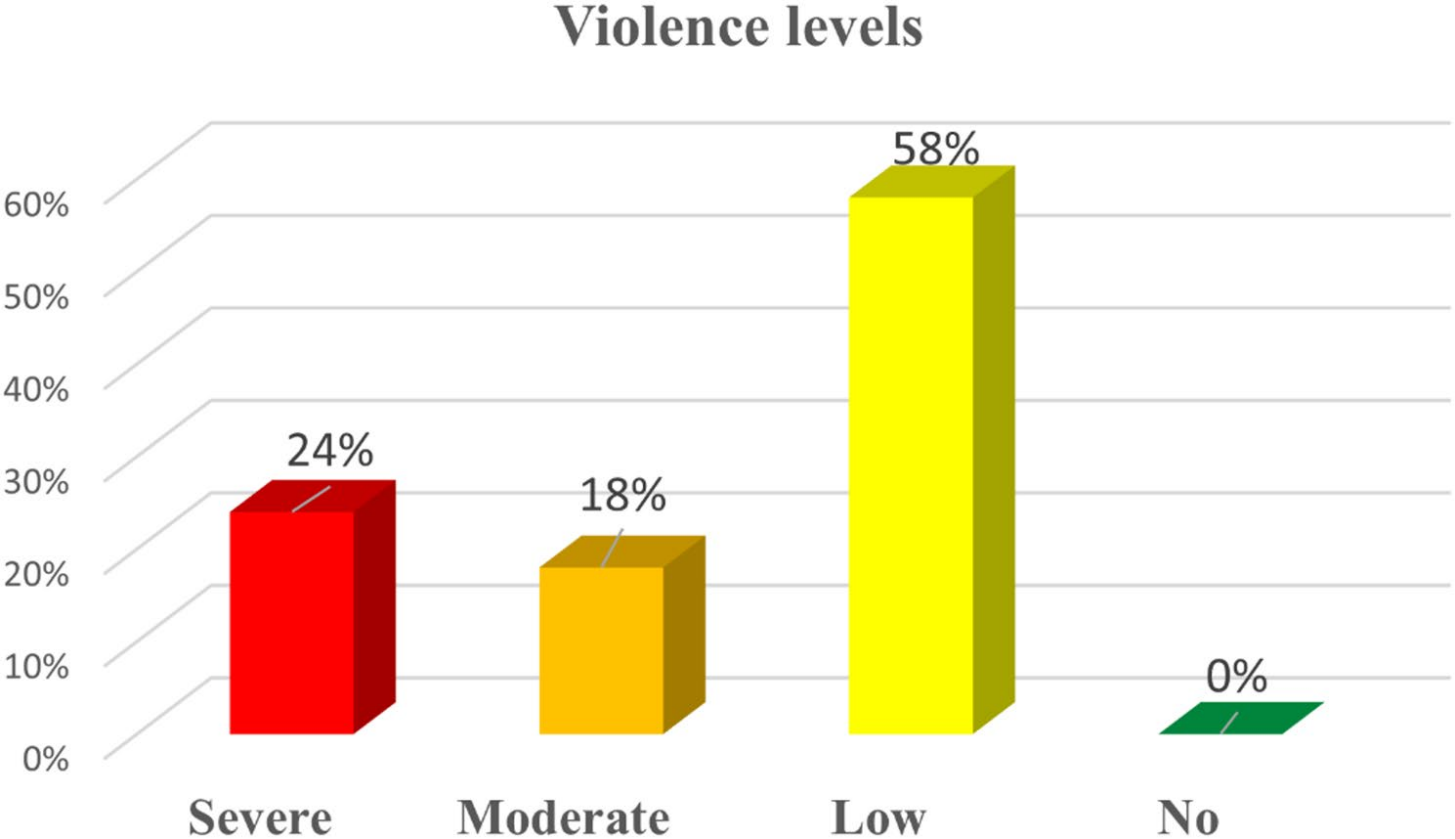
Violence levels in the sample *n*= (100). Abuse severity levels were categorized as “No violence” (score < 22), “Low” (22–50), “Moderate” (51–79), and “Severe” (80–88) based on sample distribution

As shown in Fig. 3 most mothers experienced low violence (58%), while 18% and 24% experienced moderate and severe levels, respectively. A substantial proportion of mothers were also exposed to moderate or severe violence.

Table 4 shows that higher levels of violence were significantly associated with child behavioral problems (χ², *p* =.008; *r* =.650, *p* <.001; AOR = 1.043, 95% CI: 1.003–1.084, *p* =.036) and PTSD severity (χ², *p* =.001; *r* =.648, *p* <.001; β = 0.648, *p* <.001). By contrast, the association with the mother–child relationship was negative but not significant (*r* = –.185, *p* =.066). ANOVA indicated that child age (*p* =.002) and mother’s education (*p* =.044) influenced relationship scores, while child gender and mother’s occupation were not significant. Overall, violence severity predicted adverse behavioral and PTSD outcomes, with demographic factors moderating mother–child relationship quality.

**Table 4:** Relationship between severity of violence (SVAWS) and study variables

Outcome/Analysis	Statistic	Value	*p*-value	Interpretation
Child behavior (CBCL)	Chi-square	χ² = 24.16	0.008**	Higher violence level significantly associated with more borderline and clinical behavior concerns.
	Pearson r	*r* =.650	< 0.001**	Strong positive correlation between violence and behavioral problems.
		AOR (Clinical vs. Normal) = 1.043 (95% CI: 1.003–1.084)	0.036*	Violence significantly predicted clinical-level behavior problems.
Post-traumatic stress (PTSD)	Chi-square	χ² = 25.54	0.001**	Higher violence level significantly associated with more severe PTSD.
	Pearson r	*r* =.648	< 0.001**	Strong positive correlation between violence and PTSD severity.
	Linear regression	β = 0.648 (B = 0.710, SE = 0.084)	< 0.001**	Violence strongly predicted PTSD severity.
Mother–child relationship (CPRS)	Pearson r	*r* = –.185	0.066	Negative, non-significant correlation with violence.
	Linear regression	β = –0.185 (B = –0.072, SE = 0.039)	0.066	Violence was not a significant predictor.
Child–mother relationship by demographics	ANOVA (age)	F(10,89) = 3.123	0.002**	Child age significantly affected relationship scores.
	ANOVA (education)	F(2,97) = 3.230	0.044*	Mother’s education significantly associated.

*p <.05; **p <.01

Overall, as shown in Table 4, these findings indicate that the severity of violence is significantly associated with adverse child behavioral outcomes and higher levels of PTSD symptoms, while maternal and child demographic characteristics may moderate its impact on the mother–child relationship. Interpreted through the lens of attachment theory, disrupted caregiving and reduced maternal sensitivity emerge as key mechanisms linking maternal violence exposure to child emotional and behavioral vulnerabilities.

Table 5 shows that child age and gender were significantly associated with behavior level, with behavioral problems more common among boys and older children.

**Table 5:** Relationship between Socio-Demographic factors and child behavior (CBCL) level (*N* = 100)

Variable	Normal (*n* = 40)	Borderline (*n* = 45)	Clinical Concern (*n* = 15)	*p*-value
Child age				0.017*
3–6 years	12 (30.0%)	11 (24.4%)	0 (0.0%)	
6–12 years	19 (47.5%)	24 (53.3%)	10 (66.7%)	
> 12 years	9 (22.5%)	10 (22.3%)	5 (33.3%)	
Child gender				0.002**
Male	10 (25.0%)	24 (53.3%)	10 (66.7%)	
Female	30 (75.0%)	21 (46.7%)	5 (33.3%)	
Mother age				0.417
< 25 years	1 (2.5%)	2 (4.4%)	1 (6.7%)	
25–35 years	31 (77.5%)	34 (75.6%)	12 (80.0%)	
36–40 years	7 (17.5%)	9 (20.0%)	2 (13.3%)	
> 40 years	1 (2.5%)	0 (0.0%)	0 (0.0%)	
Mother education				0.342
Preparatory	0 (0.0%)	0 (0.0%)	1 (6.7%)	
Secondary	11 (27.5%)	11 (24.4%)	0 (0.0%)	
University	29 (72.5%)	34 (75.6%)	14 (93.3%)	
Mother occupation				0.612
Housewife	23 (57.5%)	26 (57.8%)	10 (66.7%)	
Working	17 (42.5%)	19 (42.2%)	5 (33.3%)	

Chi-square test used. *p <.05; **p <.01

Table 6 shows that PTSD severity was significantly associated with child age and mother’s occupation, with higher levels among school-aged children and those with non-working mothers.

**Table 6:** Relationship between Socio-Demographic factors and Post-Traumatic stress disorder (*n* = 100)

Variable	Mild (*n* = 25)	Moderate (*n* = 40)	Moderate to Severe (*n* = 35)	*p*-value
Child age				0.005**
3–6 years	5 (20.0%)	16 (40.0%)	2 (5.7%)	
6–12 years	15 (60.0%)	9 (22.5%)	29 (82.8%)	
> 12 years	5 (20.0%)	15 (37.5%)	4 (11.5%)	
Child gender				0.230
Male	5 (20.0%)	25 (62.5%)	14 (40.0%)	
Female	20 (80.0%)	15 (37.5%)	21 (60.0%)	
Mother age				0.081
< 25 years	2 (8.0%)	2 (5.0%)	0 (0.0%)	
25–35 years	20 (80.0%)	29 (72.5%)	28 (80.0%)	
36–40 years	3 (12.0%)	8 (20.0%)	7 (20.0%)	
> 40 years	0 (0.0%)	1 (2.5%)	0 (0.0%)	
Mother education				0.328
Preparatory	0 (0.0%)	0 (0.0%)	1 (2.8%)	
Secondary	6 (24.0%)	13 (32.5%)	3 (8.6%)	
University	19 (76.0%)	27 (67.5%)	31 (88.6%)	
Mother occupation				0.033*
Housewife	10 (40.0%)	25 (62.5%)	24 (68.5%)	
Working	15 (60.0%)	15 (37.5%)	11 (31.5%)	

Chi-square test used. *p <.05; **p <.01

Table 7 shows that child age and mother’s education were significantly associated with child–mother relationship scores, while child gender and mother’s occupation were not.

**Table 7:** ANOVA of Child–Mother relationship (CPRS) by demographic factors (*n* = 100)

Variable	Source	Sum of Squares	df	Mean Square	F	Sig.
Child Age	Between Groups	1863.656	10	186.366	3.123	0.002*
	Within Groups	5311.654	89	59.682		
Mother Age	Between Groups	1067.018	19	56.159	0.736	0.772
	Within Groups	6108.292	80	76.354		
Child Gender	Between Groups	15.782	1	15.782	0.216	0.643
	Within Groups	7159.528	98	73.056		
Mother Education	Between Groups	448.063	2	224.032	3.230	0.044*
	Within Groups	6727.247	97	69.353		
Mother Occupation	Between Groups	0.415	1	0.415	0.006	0.940
	Within Groups	7174.895	98	73.213		

ANOVA used to compare means CPRS scores across demographic categories

*p <.05; **p <.01

Table 8 illustrates the associations between selected socio-demographic characteristics and key study outcomes. Analyses revealed that child age was significantly related to both behavioral problems and PTSD levels, with older children exhibiting more clinical concerns. Child gender was significantly associated with behavioral issues, as boys demonstrated more externalizing symptoms, though no significant associations were found with PTSD or the mother–child relationship. Mother’s education showed a significant positive association with the mother–child relationship, indicating a protective role, while maternal occupation was significantly related to PTSD, where unemployed mothers reported higher post-traumatic symptoms.

**Table 8:** Relationship between selected Socio-Demographic variables and study outcomes (*n* = 100)

Variable	Child Behavior (CBCL) *p*-value	PTSD Level *p*-value	Mother–Child Relationship (ANOVA) *p*-value
Child age	0.017*	0.005**	0.002**
Child gender	0.002**	0.230	0.643
Mother age	0.417	0.081	0.772
Mother education	0.342	0.328	0.044*
Mother occupation	0.612	0.033*	0.940

*Pearson’s chi-square test for categorical variables and one-way ANOVA for continuous variables*

*p <.05 = statistically significant, *p <.01 = highly significant

## Discussion

This study highlights the multifaceted impact of DV on maternal–child relationships and children’s psychological outcomes. Consistent with prior research, children exposed to maternal violence exhibited significant behavioral difficulties, aligning with findings from Egypt and the broader MENA region [42–44]. These findings suggest that higher exposure to maternal violence may disrupt the caregiver–child bond and maternal sensitivity, undermining the child’s sense of security and contributing to behavioral difficulties [45].

Domestic violence was also found to have profound consequences for maternal psychological well-being. Evidence from Egypt and the broader MENA region consistently demonstrates that women exposed to IPV are at heightened risk of depression, anxiety, and post-traumatic stress disorder (PTSD) [46, 47].

A study in Jordan similarly reported that IPV-exposed women experienced significantly higher levels of emotional distress, which compromised their capacity for sensitive caregiving [48]. These findings underscore that the impact of violence extends beyond the child, directly affecting maternal mental health.

The present study’s results align with this evidence, as increased severity of violence was significantly associated with higher PTSD levels. This suggests that maternal distress may function both as a direct outcome of IPV and as a mediating pathway through which children develop behavioral and psychological difficulties. Such evidence reinforces the need for trauma-informed services that prioritize maternal mental health as an integral component of interventions for families affected by domestic violence [44, 49].

Similarly, violence was strongly associated with post-traumatic stress disorder (PTSD) in children. This is consistent with studies from Lebanon and Egypt demonstrating that maternal IPV exposure exacerbates children’s stress responses and PTSD symptoms [46, 50]. Interpreted through attachment theory, repeated or severe exposure to violence may compromise maternal emotional availability, increasing children’s vulnerability to PTSD symptoms [51].

By contrast, the association between violence and the mother–child relationship was negative but did not reach statistical significance. Regression analysis confirmed that violence was not a significant predictor of mother–child relationship quality. Nevertheless, even a marginal negative association aligns with attachment theory, suggesting that exposure to violence may weaken maternal sensitivity and the child’s sense of security [45]. This echoes findings from Jordan, where IPV-exposed mothers demonstrated compromised parenting practices, affecting relational quality [48, 52].

Sociodemographic factors also played a role. Maternal education emerged as an important predictor of child outcomes, consistent with evidence from Egypt and other LMICs^1^ [53, 54]. Children of more educated mothers tend to show fewer behavioral problems and stronger resilience, suggesting that maternal education may buffer some of the adverse effects of DV. Conversely, maternal employment showed no significant effect on the mother–child relationship in this study, in line with mixed findings from Egyptian and regional studies [55–57].

Gender differences were also observed, with boys exhibiting more behavioral concerns than girls. This is consistent with cross-cultural findings on gender-role expectations in Egypt, where boys are often subject to harsher disciplinary practices and may externalize stress responses [58–60]. The persistence of these differences underscores the need for culturally tailored interventions that account for local norms and gender socialization patterns.

Taken together, these results emphasize that DV not only jeopardizes children’s immediate psychological well-being but also threatens the broader attachment-based caregiver–child bond. Regional studies in Egypt and neighboring countries reinforce the conclusion that maternal sensitivity and relational security are critical pathways through which violence affects children’s development [44, 46, 47, 61]. Strengthening maternal support systems and integrating trauma-informed interventions into maternal and child health services may mitigate these negative outcomes and enhance resilience [49, 62].

The findings of this study revealed significant associations between exposure to domestic violence, maternal psychological distress, and child behavioral problems. Mothers who experienced higher levels of domestic violence reported greater symptoms of post-traumatic stress disorder (PTSD), which were, in turn, associated with more behavioral difficulties among their children. These findings align with previous research suggesting that maternal mental health serves as a key mediator linking family violence and child adjustment problems [1, 63]. Although a negative relationship was observed between violence severity and mother–child relationship quality, this association was relatively weak compared to other correlations. This may suggest that the direct effects of violence on the mother–child relationship are more complex or may operate indirectly through maternal psychological distress [64].

The results underscore the importance of early identification of domestic violence and maternal distress within primary healthcare settings. Nurses are uniquely positioned to recognize early signs of family violence, provide psychosocial support, and facilitate referrals to appropriate services. Interventions that focus on improving maternal coping strategies, emotional regulation, and parenting skills may help mitigate the negative effects of violence exposure on child emotional and behavioral development [65].

The present study benefits from its Egyptian setting, offering insight into domestic violence and child behavioral outcomes within a context where the prevalence of abuse is substantive and cultural dynamics are complex. As noted, approximately one in three married women in Egypt report violence by a spouse. Yet, underreporting remains likely given social stigma and cultural factors that may normalize or obscure certain forms of abuse. The way in which violence is defined culturally and the degree to which mothers perceive, label, or disclose it—may influence both measurement and interpretation of findings. For instance, psychological or emotional violence may be minimized or unrecognized, hindering disclosure and biasing prevalence estimates. These considerations highlight the importance of contextualizing the findings: nursing interventions in Egypt must attend not only to the presence of violence, but also to how violence is understood, disclosed and addressed within families, communities and primary care systems.

### Study strengths

This study has several strengths that enhance the value of its findings. First, it provided a comprehensive assessment by examining multiple outcomes; child behavior, post-traumatic stress symptoms, and mother–child relationship, offering a holistic understanding of the impact of domestic violence. Second, the use of standardized and validated instruments, including the SVAWS, CBCL, and CPRS, strengthened the reliability and comparability of the results. Third, interpreting the findings within the framework of attachment theory offered a clear lens for understanding the mechanisms through which maternal violence exposure may influence child and relational outcomes. Finally, the study contributes contextual relevance by generating region-specific evidence from Egypt and the broader Middle Eastern context, thereby addressing a critical gap in the literature.

### Study limitations and future directions

Several limitations should be considered when interpreting the findings of this study. First, the descriptive correlational design limits the ability to infer causality among the examined variables. Although significant associations were identified, causal relationships cannot be established. Future studies employing longitudinal or experimental designs are recommended to explore the underlying mechanisms linking domestic violence, maternal mental health, and child behavioral outcomes.

Second, the use of a convenience sample of 100 mothers from a single Maternal and Child Health Center in Assiut limits the generalizability of the results. The findings may not represent mothers and children from other regions or socioeconomic backgrounds across Egypt. Future research should include larger, more diverse samples using stratified random sampling across rural and urban settings to enhance external validity.

Third, all key variables domestic violence exposure, mother–child relationship quality, and child behavioral problems were assessed through maternal self-reports. This reliance on a single source may introduce common method bias, as maternal perceptions and emotional states could have influenced the reported data [66]. Future studies should incorporate multiple informants (e.g., fathers, teachers, or independent observers) and objective behavioral assessments to improve data validity.

Fourth, the relationship between violence severity and mother–child relationship quality was weaker than the correlations involving maternal PTSD and child behavior problems. This pattern suggests that maternal psychological distress, such as PTSD or depression, may mediate the association between violence exposure and mother–child interaction [67]. Future studies could employ mediation or path analysis models to clarify these indirect pathways.

Fifth, the study utilized the Severity of Violence Against Women Scale (SVAWS), which provides an overall composite score of violence severity without differentiating between physical, psychological, or economic forms. Consequently, it was not possible to examine how each type of violence uniquely influences children’s internalizing and externalizing behaviors. Future research should employ detailed subscale or separate instruments to identify which forms of violence are the strongest predictors of specific child outcomes.

Additionally, although the SVAWS was culturally adapted for use in this study, the shortened 22-item version has not undergone full psychometric validation within the Egyptian context, which may limit comparability with studies using the original scale. Finally, while the study offers valuable insights from the Egyptian cultural context, findings should be interpreted within local social norms and definitions of violence. Cultural beliefs surrounding family privacy, gender roles, and stigma may influence how domestic violence is perceived and reported [68]. Future research should integrate recent national data on domestic violence prevalence and explore how cultural and socioeconomic factors affect women’s disclosure and coping behaviors. Such culturally grounded evidence is essential for developing effective nursing interventions and family-centered prevention strategies.

### Recommendations

#### For children

- Engagement in schooling is a recognized protective factor for adverse childhood events. Regular school attendance is particularly important for children, as it offers a vital pathway for academic and social success.
- Education sector needs to explore evidence-based and innovative strategies to support children who experience family and DV.
- Government initiatives that address the cycle of DV must also be developed in consultation with community and its impact on children’s school engagement.

#### For women

- Social support and proactive coping styles may reduce the likelihood that PTSD will develop.
- Cognitive processing therapy is an evidence-based 12-session manualized treatment for PTSD.

## Supplementary Information

Supplementary Material 1.

## Data Availability

The datasets generated during and/or analyzed during the current study are available from the corresponding author on reasonable request.

## Footnotes

1
*Low- and middle-income countries*.
