# Supplementary material for: Domestic violence’s impact on maternal–child relationship and child behavior: a nursing study from Egypt
Source: BMC Psychol. 2026 Jan 20;14:85. doi: 10.1186/s40359-025-03763-0 (PMC12821966; doi:10.1186/s40359-025-03763-0)
Supplement: Supplementary file 1 — Supplementary Material 1. [file 40359_2025_3763_MOESM1_ESM.pdf]

The Severity of Violence against Women Scale (SVAWS)

no	**Items**	Never (1)	once (2)	a few times (3)	many times (4)
1	**How often has your partner:**<br><br>Hit or kicked a wall, door, or furniture				
2	Thrown, smashed, or broke something				
3	Drove-dangerously with you				
4	Throwing something at you				
5	Made threatening gestures or facial expressions toward you				
6	Waggled his or her finger at you				
7	Acted like a bully toward you				
8	Threatened to hurt or destroy things you love				
9	Destroyed something that belongs to you				
10	Threatened to kill you				
11	Threatened you with a weapon or knife				
12	Threatened someone you love				
13	Shocked or grabbed you suddenly or forcefully				
14	Pulled your hair				
15	Holed you, held you in place				
16	Twisted your arm, hit you, or bit you				
17	Slapped you in the face or head				
18	Slapped you with the palm of his or her hand or the back of his or her hand				
19	Hit you with an object				
20	Punched, kicked, or stomped on you				
21	Choked or burned you with something				
22	Forced you to have sex against your will				

Post-Traumatic Stress Scale for Family Violence (PTSDS)

no	**Items**	**Not at all (0)**	**A little bit (1)**	**Moderately (2)**	**Quite a bit (3)**	**Extremely(4)**
1	**In the past month, how much were you bothered by:** Repeated, disturbing, and unwanted memories of the stressful experience?					
2	Repeated, disturbing dreams of the stressful experience?					
3	Suddenly feeling or acting as if the stressful experience were actually happening again (as if you were actually back there reliving it)?					
4	Feeling very upset when something reminded you of the stressful experience?					
5	Having strong physical reactions when something reminded you of the stressful experience (for example, heart pounding, trouble breathing, sweating)?					
6	Avoiding memories, thoughts, or feelings related to the stressful experience?					
7	Avoiding external reminders of the stressful experience (for example, people, places, conversations, activities, objects, or situations)?					
8	Trouble remembering important parts of the stressful experience?					
9	Having strong negative beliefs about yourself, other people, or the world (for example, having thoughts such as: I am bad, there is something seriously wrong with me, no one can be trusted, and the world is completely dangerous)?					
10	Blaming yourself or someone else for the stressful experience or what happened after it?					
11	Having strong negative feelings such as fear, horror, anger, guilt, or shame?					
12	Loss of interest in activities that you used to enjoy?					

13	Feeling distant or cut off from other people?					
14	Trouble experiencing positive feelings (for example, being unable to feel happiness or have loving feelings for people close to you)?					
15	Irritable behavior, angry outbursts, or acting aggressively?					
16	Taking too many risks or doing things that could because you harm?					
17	Being "super alert" or watchful or on guard?					
18	Feeling jumpy or easily startled?					
19	Having difficulty concentrating?					
20	Trouble falling or staying asleep?					

Child–Parent Relationship Scale (CPRS)

no	Items	Definitely does not apply(0)	Not reall y(1)	Neutral, not sure(2)	Applies somewhat (3)	Definitely applies (4)
1	**I share a warm, affectionate relationship with my child**					
2	**He looks neat and my child always struggles with some people**					
3	**If my child is upset, he will ask me for affection**					
4	**My child never feels physical affection or touch from me**					
5	**My child is able to finish me**					
6	**When I praise my child, he radiates pride**					
7	**My child shares information about retail**					
8	**My child gets angry easily**					
9	**It is easy for me to be accommodating with what my child is feeling**					
10	**My child gets angry or resistant after being disciplined**					
11	**Dealing with my child drains my energy**					
12	**When my child is in a bad mood, he knows we will have a long and difficult time**					
13	**My child's feelings can be unexpected or can fall out**					
14	**My child is restrained or treated with me**					
15	**My child shares his feelings and experiences with me by expressing**					

Child Behavior Checklist (CBCL)

no	**Items**	Not true (0)	Somewhat true or sometimes true (1)	Very true or often true (3)
1	Aches or pains (without medical cause; do not include stomach or headaches)			
2	Acts too young for age.			
3	Afraid to try new things.			
4	Avoids looking others in the eye.			
5	Can't concentrate, can't pay attention for long			
6	Can't sit still, restless, or hyperactive			
7	Can't stand having things out of place			
8	Can't stand waiting; wants everything now			
9	Chews on things that aren't edible			
10	Clings to adults or too dependent			
11	Constantly seeks help			
12	Constipated, doesn't move bowels (when not sick)			
13	Cries a lot			
14	Cruel to animals			
15	Defiant			
16	Demands must be met immediately			
17	Destroys his/her own things			
18	Destroys things belonging his/her family or other children			
19	Diarrhea or loose bowels (when not sick)			
20	Disobedient			
21	Disturbed by any change in routine			
22	Doesn't want to sleep alone			
23	Doesn't answer when people talk to him/her			
24	Doesn't eat well			
25	Doesn't get along with other children			
26	Doesn't know how to have fun; acts like a little adult			
27	Doesn't seem to feel guilty after misbehaving			
28	Doesn't want to go out of home			

29	Easily frustrated			
30	Easily jealous			
31	Eats or drinks things that are not food– don't include sweets (describe):			
32	Fears certain animals, situations, or places (describe):			
33	Feelings are easily hurt			
34	Gets hurt a lot, accident-prone			
35	Gets in many fights			
36	Gets into everything			
37	Gets too upset when separated from parents			
38	Has trouble getting to sleep			
39	Headaches (without medical cause)			
40	Hits others			
41	Holds his/her breath			
42	Hurts animals or people without meaning to			
43	Looks unhappy without good reason			
44	Angry moods			
45	Nausea, feels sick (without medical cause)			
46	Nervous movements or twitching (describe):			
47	Nervous, highstrung, or tense			
48	Nightmares			
49	Overeating			
50	Overtired			
51	Shows panic for no good reason			
52	Painful bowel movements (without medical cause)			
53	Physically attacks people			
54	Picks nose, skin, and other parts of body (describe):			
55	Plays with own sex parts too much			
56	Poorly coordinated or clumsy			
57	Problems with eyes (without medical cause) (describe):			
58	Punishment doesn't change his/her behavior			
59	Quickly shifts from one activity to another			
60	Rashes or other skin problems (without medical causes)			
61	Refuses to eat			

62	Refuses to play active games			
63	Repeatedly rocks head or body			
64	Resists going to bed at night			
65	Resists toilet training (describe):			
66	Screams a lot			
67	Seems unresponsive to affection			
68	Self-conscious or easily embarrassed			
69	Selfish or won't share			
70	Shows little affection toward people			
71	Shows little interest in things around him/her			
72	Shows too little fear of getting hurt			
73	Too shy or timid			
74	Sleeps less than most kids during day and/or night (describe):			
75	Smears or plays with bowel movements			
76	Speech problem (describe):			
77	Stares into space or seems preoccupied			
78	Stomachaches or cramps (without medical cause)			
79	Rapid shifts between sadness and excitement			
80	Strange behavior (describe):			
81	Stubborn, sullen, or irritable			
82	Sudden changes in mood or feelings			
83	Sulks a lot			
84	Talks or cries out in sleep			
85	Temper tantrums or hot temper			
86	Too concerned with neatness or cleanliness			
87	Too fearful or anxious			
88	Uncooperative			
89	Underactive, slow moving, lacks energy			
90	Unhappy, sad, or depressed			
91	Unusually loud			
92	Upset by new people or situations (describe):			
93	Vomiting, throwing up (without medical cause)			
94	Wakes up often at night			
95	Wanders away			
96	Wants a lot of attention			
97	Whining			
98	Withdrawn, doesn't get involved with others			

99	Worries			
100	Please write in any problems the child has that were not listed above:			
